# Femoral Shaft Fractures in Children: Exploring Treatment Outcomes and Implications

**DOI:** 10.7759/cureus.46336

**Published:** 2023-10-01

**Authors:** Muhammad Maaz G Kakakhel, Nouman Rauf, Sultan Ahmad Khattak, Pritha Adhikari, Zahid Askar

**Affiliations:** 1 Trauma and Orthopaedics, Liverpool University Hospitals NHS Foundation Trust, Liverpool, GBR; 2 Orthopedic Surgery, Khyber Girls Medical College, Peshawar, PAK; 3 Orthopedics, Hayatabad Medical Complex Peshawar, Peshawar, PAK; 4 Orthopedics, Kingston Hospital, Kingston, GBR; 5 Medicine, Nepalgunj Medical College, Kohalpur, NPL; 6 Trauma and Orthopaedics, Khyber Teaching Hospital, Peshawar, PAK

**Keywords:** quality of life, accidents, children, hip spica casting, femur shaft injuries, femur shaft fractures

## Abstract

Introduction: Femoral shaft fractures significantly impact children and families, posing a significant challenge for pediatric patients. The prevalence of limb shortening in femur shaft fractures treated with hip spica casting in our group, however, has not been the subject of many recent investigations.

Aims: By comparing the prevalence of limb shortening to various age groups and common pediatric injury patterns, this research seeks to close this information gap.

Methods: This research, which lasted six months and was done at the Orthopedics Unit of Khyber Teaching Hospital Peshawar, Pakistan, included 129 children with closed femur shaft fractures who were between the ages of one and six. Clinical assessments, radiological examinations, and hip spica casting, all supervised by experienced orthopedic surgeons, were carried out. Senior postgraduate trainees oversaw the study's findings.

Results: The results unveiled key insights into the study population. Among the findings, 33% (n=43) of the children were aged one to three years, while 67% (n=86) fell within the three to six years age range. Gender distribution revealed that 72% (n= 93) were male. In terms of mechanism, 22% (n=28) of fractures were attributed to road traffic accidents, 69% (n=89) were the result of falls, and 5% (n=12) were due to other causes. Notably, 19% (n=29) of the children exhibited limb shortening.

Conclusion: This study contributes significantly to the understanding of femur shaft fractures in children, shedding light on their complex dynamics. The study enhances our understanding of pediatric femur shaft fractures. We found that 19% of children exhibited limb shortening, underscoring the need for targeted treatment strategies. These insights can significantly improve patient care and treatment protocols for this challenging condition, benefiting both children and their families.

## Introduction

Femoral shaft fractures, though relatively infrequent, can present a considerable challenge when they occur in children across all age groups. Accounting for approximately 1.6% of all childhood fractures, these injuries exert a substantial physical, emotional, and psychological impact not only on the affected child but also on their family [1]. The intricacies of treating such fractures are multifaceted, with considerations ranging from the patient's age to the unique fracture pattern exhibited.

While several studies have contributed to our understanding of femoral shaft fractures in diverse populations, there remains a significant knowledge gap within our specific demographic context. Surprisingly, no comprehensive study has been conducted within our region over the past five years [2], leaving a crucial gap in our understanding of the dynamics and outcomes related to these fractures. This impending research undertaking seeks to bridge this knowledge gap and offer the most up-to-date insights into the prevalence of limb shortening resulting from femur shaft fractures in children treated with hip spica casting [3,4]. By delving into this unexplored territory, this study aspires to not only contribute vital information to our local medical community but also serve as a resource for health professionals globally. The outcome of this research endeavor will be instrumental in refining treatment strategies, fostering better patient care, and shaping recommendations for optimal fracture management. Beyond immediate clinical applications, the findings of this study are poised to lay the foundation for future scholarly work [5], potentially enriching the body of knowledge surrounding femoral shaft fractures in children.

As this important exploration journey commences, the anticipated results hold the promise of empowering medical practitioners with evidence-based insights that can tangibly enhance the lives of young patients and their families [6]. The dissemination of these findings across the medical landscape will undoubtedly contribute to a more comprehensive understanding of femoral shaft fractures in children, fostering a collaborative environment of learning and improved care [7].

## Materials and methods

Study setting: This research was conducted at Khyber Teaching Hospital Peshawar, Pakistan.

Duration of study: The trial lasts for six months, from February 12, 2020, to August 12, 2020.

Study design: The study design was a descriptive study.

Sample size: Following the WHO methodology for sample size determination, we arrived at a total sample size of 129. This calculation considered a 22% incidence rate of shortened limbs in children treated with hip spica casting for femur shaft fractures, a 95% confidence level, and an 8% margin of error.

Sampling technique: The method of non-probability sequential sampling was employed to acquire samples.

Sample selection

Inclusion criteria: The research included all children between the ages of one to six years old, both genders (male/female), who presented with a closed proximal, middle, or distal femur shaft fracture on one side and a normal side.

Exclusion criteria: The study excluded all children who had pathological fractures linked to diseases such as osteomyelitis, rickets, tumors or cysts, and osteogenesis imperfecta, as determined by the history, clinical examination, and radiological examination. These conditions act as confounders and can skew the findings.

Data collection procedure: The study obtained ethical approval and enrolled children with femur shaft fractures on one side and a normal side from Khyber Teaching Hospital Peshawar's Emergency/OPD and Orthopedics Department. Parental consent was secured upon admission. Thorough history, clinical, and radiological exams confirmed the fractures. Experienced orthopedic surgeons performed hip spica casting within the first 24-48 hours of fracture confirmation, followed by daily supervised assessments of cast integrity and patient compliance. The assessment for femur shortening was conducted precisely at the time of cast removal, based on a well-defined formula. Senior postgraduate trainees, overseen by a Consultant Orthopedics specialist, conducted assessments. Limb shortening, defined as a 2 cm+ difference compared to the unaffected side, was measured from the anterior superior iliac spine to the medial malleolus. Data on age, gender, and fracture details were collected, adhering to strict exclusion criteria.

Data analysis: The statistical program Statistical Product and Service Solutions (SPSS) (version 23; IBM SPSS Statistics for Windows, Armonk, NY) was used to input all the data from the proforma and apply descriptive statistics. For continuous variables, including age, fracture duration, pre-operative limb length, and post-operative limb length, the mean and standard deviation were calculated. Limb length measurements were purely clinical, based on apparent limb length differences, without the use of pre-operative or post-operative X-ray assessments. For categorical variables, including gender, kind of trauma, location of femur shaft fracture, and limb shortening, frequency and % were calculated. Limb shortening was stratified according to age, gender, the length of the fracture, the kind of trauma, and the location of the femur shaft fracture to observe the effects. The post-stratification chi-square test was used, with a P value of 0.05 being deemed significant. Tables and charts were used to show all of the data.

Ethical statement

Ethical approval (417/ADR/KMC, Dated: 3/5/2019) has been granted by the Institutional Research and Ethical Review Board (IREB) at Khyber Medical College, Peshawar, Pakistan for this research.

## Results

Based on our research data, within a sample of 129 youngsters, as indicated in Table 1, 43 (33%) were in the age range of one to three years, and 86 (67%) were in the age range of three to six years. Furthermore, 36 children (28%) were female, while 93 (72%) were male.

**Table 1:** Age Distribution Gender Distribution SD: standard deviation; P<0.05 is significant.

Age	Frequency	Percentage	Mean±SD	P
1-3 years	43	33%	64.5±30.40	0.205
3-6 years	86	67%		
Total	129	100%		
Gender				
Male	93	72%	-	0.265
Female	36	28%		
Total	129	100%		

The 21 (16%) and 108 (84%) of children sustained fractures that were treated more than 48 hours later (Table 2). Road traffic accidents (RTA) caused 28 (22%) children to fracture, falls caused 89 (69%) children to fracture, and other causes caused 12 (5%) children to fracture. In Table 2, proximal femoral shaft fractures occurred in 71 (55%) children, intermediate femoral shaft fractures in 53 (41%) children, and distal femoral shaft fractures in five (4%).

**Table 2:** Femoral Shaft Fracture Characteristics SD: standard deviation; N: sample size; %: percentage; Mean: mean value; ±: Plus-minus sign indicating the range around the mean; RTA: road traffic accident P value is considered significant when p<0.05.

Duration	Frequency	Percentage	Mean±SD	P
≤ 2 days	108	84%	86±57.23	0.121
> 2 days	21	16%		
Total	129	100%		
Type of trauma				
RTA	28	22%	43±40.63	0.208
Fall	89	69%		
Others	12	9%		
Total	129	100%		
Site				
Proximal	71	55%	43±34.11	0.161
Middle	53	41%		
Distal	5	4%		
Total	129	100%		

Table 3 provides a comprehensive overview of limb shortening case stratification by age, including both frequencies and percentages. Leg shortening was observed in 25 out of 129 cases. Nineteen percent of those with shortening in the study were between the ages of one and three years, accounting for eight cases. The remaining 81%, equivalent to 17 cases, fell within the age range of three to six years. The statistical analysis produced a P value of 0.8748, which was non-significant. In cases where there was no length reduction, 81% of the cases (n=75) were between the ages of one to three years, while 19% (n=29)were between the ages of three to six years. Furthermore, the P value of 0.9907 obtained was found to be non-significant. According to the data, there are no significant age differences in the prevalence of limb shortening.

**Table 3:** Age-Related Stratification of Limb Shortening P value is considered significant when p<0.05.

Shortening of Limb	Frequency	Percentage	1-3 years	3-6 years	Total	P value
Yes	25	19%	8	17	25	0.8748
No	104	81%	35	69	104	
Total	129	100%	43	86	129	
Yes	25	19%	18	7	25	0.9907
No	104	81%	75	29	104	
Total	129	100%	93	36	129	

Table 4 displays a comprehensive analysis of limb shortening, taking into account the duration of the condition (two days versus > two days), the type of trauma (RTA, fall, other), and the specific site of the injury (proximal, middle, distal). The table further offers valuable information about the statistical properties of the data. The numbers denoted as “Mean±SD” indicate the mean number of events, as well as the associated variability represented by the standard deviation, across various groups. As an example, the first set of statistics (54±46.67, 10±9.19, 14±11.31) pertains to occurrences transpiring over a span of two days and classifies them according to their respective types, namely, RTA, falls, and other events. The second set of data (44±38.89, 6±5.67) corresponds to occurrences that have been categorized based on their kind. The final set, denoted as 35±30.40, represents instances that have been categorized based on their closeness. Moreover, the “P” values provide insights into the statistical significance of observed disparities across different incident groups. These values are often used to ascertain if the observed disparities between groups may be attributed to chance or whether they possess substantive significance. In the present scenario, the p values (0.97, 0.35, 0.33, 0.35, 0.374, 0.347, 0.36, 0.34) did not indicate significant outcomes of statistical tests conducted to compare various occurrence categories.

**Table 4:** Stratification of Limb Shortening: Duration, Trauma Type, and Site Analysis SD: standard deviation; road traffic accidents (RTAs) P value is considered significant when p<0.05.

Variables	≤ 2 days	> 2 days	Total	RTA	Fall	Other	Total	Proximal	Middle	Distal	Total
Yes	21	4	25	6	17	2	25	14	10	1	25
No	87	17	104	22	72	10	104	57	43	4	104
Total	108	21	129	28	89	12	129	71	53	5	129
Mean±SD	54±46.67	10±9.19		14±11.31	44±38.89	6±5.67		35±30.40	26±23.33	2±2.12	
P	0.97	0.35		0.33	0.35	0.374		0.347	0.36	0.34	

## Discussion

Children of all ages are vulnerable to femoral shaft fractures, accounting for 1.6% of pediatric fractures and impacting both the child and their family. Treatment depends on age and fracture pattern. Incidence follows a bimodal distribution with peaks in infancy and mid-adolescence [8,9]. Simple falls or twisting cause most fractures in young kids, while non-ambulatory children face intentional maltreatment in up to 80% of cases [10]. High-energy injuries, such as car accidents, are common among teens. Earlier, treatment involved three to 12 weeks of spica casting, leading to long hospital stays and immobility. Newer options offer swift recovery and shorter stays [11]. This study echoes pediatric orthopedics trends, considering age and mechanisms. Falls dominate in young kids, while accidents prevail in teens [10]. Evolving treatments emphasize quicker recovery and enhanced patient experiences [11]. According to our research, out of 129 youngsters, 43 (33%) were between the ages of one and three, and 86 (67%) were between the ages of three and six. A total of 36 children (28%) were female, and 93 (72%) were male. Another similar study included a total of 25 children, with a mean age of 3.9 years and a distribution of 16 (64%) male, and nine (36%) female, who were treated with an initial hip spica cast. They were between the ages of 1 and 8. The majority (n=18, 72%) were children under five [12].

In the current study, 21 children (16%) suffered fractures that lasted longer than two days, while 108 children (84%) suffered fractures that did not last longer than two days. RTA caused 28 (22%) children to fracture, falls caused 89 (69%) children to fracture, and other causes caused 12 (5%) children to fracture. Flinck et al. [12] showed that proximal femoral shaft fractures occurred in 71 (55%) children, intermediate femoral shaft fractures in 53 (41%) children, and distal femoral shaft fractures in five (4%). Additionally, 104 (81%) children lacked shortening limbs, compared to 25 (19%) youngsters who did. A total of 22 (88%) of the children were tracked through the removal of the spica cast and finished the trial. After the third week and onward, three (13%) children were unable to attend a follow-up appointment. In 15 patients (60%), the mechanism of injury was a fall from a height; in six (24%) cases, it was an RTA; and in four (16%) cases, it was a heavy item falling on a limb.

The average spica cast immobilization time was 5.3 weeks, although it might have been as long as six weeks. Four (16%) children had the spica cast wedged to correct angulation, and five (20%) children suffered soakage and breaking of the spica in the second week. Shortening varied from 0.3 cm to 2 cm in seven (31% of the children) at the time of cast removal after the fracture had healed [12]. There was no evidence of the shattered limb extending or overgrowing. Three children (13%) had angulation (10-15 degrees in the front and 5-10 degrees in the side). All patients had mild to moderate acute knee joint stiffness once the spica was removed, although this stiffness resolved completely after a brief course of home exercise programs. Three children (13%) had minor skin deterioration [13].

Another research found that 2,805 individuals had FSFs; 69% of them had surgery, and 31% underwent nonsurgical treatment. In both ISS groups, mortality was greater with nonsurgical treatment. The duration of hospitalization increased in the surgically treated groups as the surgical delay increased [14]. Repair within two to four days was linked with the lowest mortality and shortest hospitalization in patients with an ISS > or = 15, while repair within one day was associated with a tendency towards greater mortality and longer hospitalization. Fourteen patients (22%) had limb length discrepancies (0.5 cm in four, 1 cm in eight, 1.5 cm in two) [15].

Limitations of the study

There are several limitations associated with the research being conducted on femoral shaft fractures in pediatric patients. Several factors may impact the generalizability of the study, including its limited period of six months and the fact that it was done just at the Orthopedics Department of Khyber Teaching Hospital, Peshawar, Pakistan. The limited sample size of 129 may provide limitations on the generalizability of the findings. The study has a specific emphasis on closed femur shaft fractures that are treated with hip spica casting, hence limiting the scope of the research. Furthermore, there is less research on the long-term effects that extend beyond the duration of the casting phase. Notwithstanding these limitations, the study enhances comprehension of pediatric femoral shaft fractures and offers valuable perspectives for enhancing treatment strategies and patient care.

## Conclusions

This research sheds light on the complex terrain of pediatric femoral shaft fractures, highlighting the many age groups and damage processes involved. Traditional treatment strategies have changed, favoring early mobilization and shorter hospital stays, and are now adapted to certain age-related trends. Comparisons with other research highlight the significance of tailored techniques and the best time for surgery. According to the results of our research, children who had hip spica casting for femur shaft fractures experienced limb shortening in 19% of the cases. Collectively, these results provide vital information that healthcare professionals may use to manage these fractures more skillfully, improving patient outcomes and quality of life.
